# Evaluation of Drug Blood-Brain-Barrier Permeability Using a Microfluidic Chip

**DOI:** 10.3390/pharmaceutics16050574

**Published:** 2024-04-23

**Authors:** Jung Yoon Yang, Dae-Seop Shin, Moonkyu Jeong, Seong Soon Kim, Ha Neul Jeong, Byung Hoi Lee, Kyu-Seok Hwang, Yuji Son, Hyeon-Cheol Jeong, Chi-Hoon Choi, Kyeong-Ryoon Lee, Myung Ae Bae

**Affiliations:** 1 Therapeutics & Biotechnology Division, Korea Research Institute of Chemical Technology, Daejeon 34114, Republic of Korea; yjy1608@krict.re.kr (J.Y.Y.); dsshin@krict.re.kr (D.-S.S.); kimss@krict.re.kr (S.S.K.); skyvwv@krict.re.kr (H.N.J.); bnhlee@krict.re.kr (B.H.L.); kshwang@krict.re.kr (K.-S.H.); yjson@krict.re.kr (Y.S.); 2 Department of Bioengineering, University of Science and Technology, Daejeon 34113, Republic of Korea; jmk@kribb.re.kr; 3 Laboratory Animal Resource & Research Center, Korea Research Institute of Bioscience and Biotechnology, Cheongju 28116, Republic of Korea; guscjf6356@kribb.re.kr; 4 Department of Medicinal Chemistry and Pharmacology, University of Science and Technology, Daejeon 34113, Republic of Korea; 5 Department of Radiology, Chungbuk National University Hospital, Chungbuk National University College of Medicine, Cheongju 28644, Republic of Korea; chihoonc@chungbuk.ac.kr

**Keywords:** BBB, microfluidic chip, physiologically based pharmacokinetic modeling

## Abstract

The blood-brain-barrier (BBB) is made up of blood vessels whose permeability enables the passage of some compounds. A predictive model of BBB permeability is important in the early stages of drug development. The predicted BBB permeabilities of drugs have been confirmed using a variety of in vitro methods to reduce the quantities of drug candidates needed in preclinical and clinical trials. Most prior studies have relied on animal or cell-culture models, which do not fully recapitulate the human BBB. The development of microfluidic models of human-derived BBB cells could address this issue. We analyzed a model for predicting BBB permeability using the Emulate BBB-on-a-chip machine. Ten compounds were evaluated, and their permeabilities were estimated. Our study demonstrated that the permeability trends of ten compounds in our microfluidic-based system resembled those observed in previous animal and cell-based experiments. Furthermore, we established a general correlation between the partition coefficient ($K_{p}$) and the apparent permeability ($P_{app}$). In conclusion, we introduced a new paradigm for predicting BBB permeability using microfluidic-based systems.

## 1. Introduction

The BBB is a selective semipermeable membrane in the brain composed of selective tight junctions, which cordon off the central nervous system (CNS). It protects the brain xenobiotic compounds. The human BBB is composed of microvascular endothelial cells (ECs), pericytes, astrocytes, tight junctions, and a basal membrane [1,2]. Astrocytes and pericytes support the barrier function and interact with endothelial cells to maintain and control vascular integrity under physiological and pathological conditions [3]. Therefore, designing neuropharmaceuticals with high BBB permeability is challenging. The low success rate of CNS drugs is a result of insufficient CNS exposure because of an inability to cross the BBB. Only small, lipid-soluble molecules of molecular weight <400 Da can cross the BBB; most macromolecules cannot. This physiological hurdle stops the development of 95% of drugs intended to treat neurological diseases. Small hydrophilic and lipophilic molecules can be passively transported across the BBB [4]. The ability of a drug to across the BBB is related to its physicochemical or pharmacokinetic properties, including absorption, distribution, metabolism, and excretion (ADME) [5].

Most in vitro models of the BBB are the two-dimensional (2D) Transwell systems. ECs are cultured on permeable membranes in cell-culture plates, thereby enabling monitoring of barrier permeability. However, Transwell models lack extracellular matrix (ECM) and fluid flow and so do not accurately model BBB microvessels. In contrast, the microfluidic system uses a variable three-dimensional (3D) structure to provide continuous nutrients and oxygen to cells through media perfusion to closely mimic the environment in humans [6]. Animal models, although more representative of humans than an in vitro system, have limitations in terms of detection methods and access to tissues; importantly, the results often vary from those in humans [7]. As a result of cost and ethical issues, and to reduce the use of animals in research, there have been efforts to develop 3D in vitro models, such as the organ-on System [8,9].

Microfluidics have recently been seen in a growing number of applications in the biology, chemistry, environmental, and biomedical fields [10]. Recently, microphysiological systems have enabled faster drug development, effective drug selection at lower risk, and effective drug production in human models [11]. To be exact, Organ-on-a-chip is a multi-channel microfluidic device compatible with cell cultures similar to the physical and physiological functions of a specific organ, overcoming limited resources available for preclinical trials for drug screening and delivery in physiologically related mechanical environments [12,13,14]. We introduce BBB permeability by analyzing chemicals through LC-MS/MS in the microfluidic system human BBB-on-a-chip. Two aspects will be covered: (1) in vitro models of BBB and characterization, and (2) BBB permeability using modeling predictions.

Test models using microfluidic chips can simulate the physical stimuli within the human body by implementing shear stress on the BBB. By simultaneously cultivating various cells that make up the BBB, these models enable the interaction between cells, reproducing functions similar to the physiological BBB. Recent advances in organ-on-a-chip technology have been reported to provide the ability to recapitulate the microenvironment of the BBB [15,16]. These BBB chips can be applied to construct various brain disease models in an environment similar to the human body and have been reported to be applicable to CNS drug development [17]. In this study, well-validated microfluidic chips were used to investigate the structure and functionality of the BBB [18]. Utilizing the established BBB chip, our study aimed to predict the blood-brain-barrier permeability of drugs in a manner similar to the human body during the preclinical stages of drug development. This was achieved by selecting substances previously reported for their BBB permeability and establishing a test method for drug BBB permeability.

## 2. Materials and Methods

### 2.1. Chemicals

To assess BBB permeability, we used caffeine (CAF), carbamazepine (CBZ), desipramine (DES), loperamide (LPM), cetirizine (CET), vincristine (VIN), nefazodone (NZD), donepezil (DPZ), and simvastatin (SIM) (all from Sigma-Aldrich). LP533401 (LP) was synthesized by the Center for Medicinal Chemistry at the Korea Research Institute of Chemical Technology (KRICT).

### 2.2. Cell Culture

Immortalized human brain microvascular endothelial cells (hBMEC; #1000, ScienCell, Carlsbad, CA, USA), human brain vascular pericytes (HBVP; #1200, ScienCell), and human astrocytes (NHA; CC-2565, Lonza, Basel, Switzerland) were maintained in endothelial cell medium (#1001, ScienCell), pericyte medium (#1201, ScienCell), and astrocyte medium (CC-3186, Lonza), respectively. Cells were cultured at 37 °C in a humidified chamber with an atmosphere of 95% air and 5% CO_2_.

### 2.3. BBB-on-a-Chip Assembly in the Device

**Figure 1:**
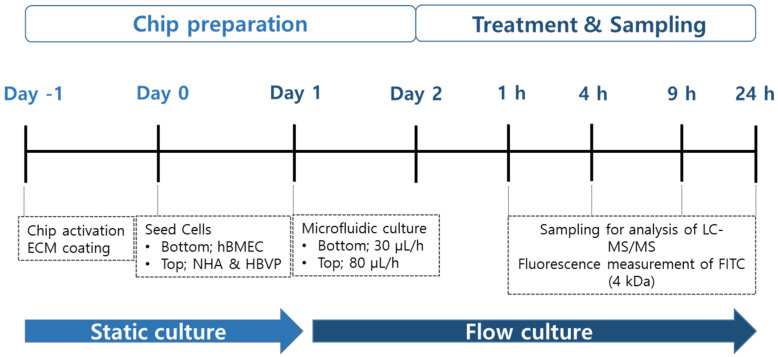
Functional validation of the in vitro human blood-brain-barrier (BBB) model. Timeline for seeding and permeability test of BBB-on-a-chip of human BBB cells. The colors of the chip preparation (light blue) and sampling (dark blue) were classified, and the colors were classified according to the culture system.

**Figure 2:**
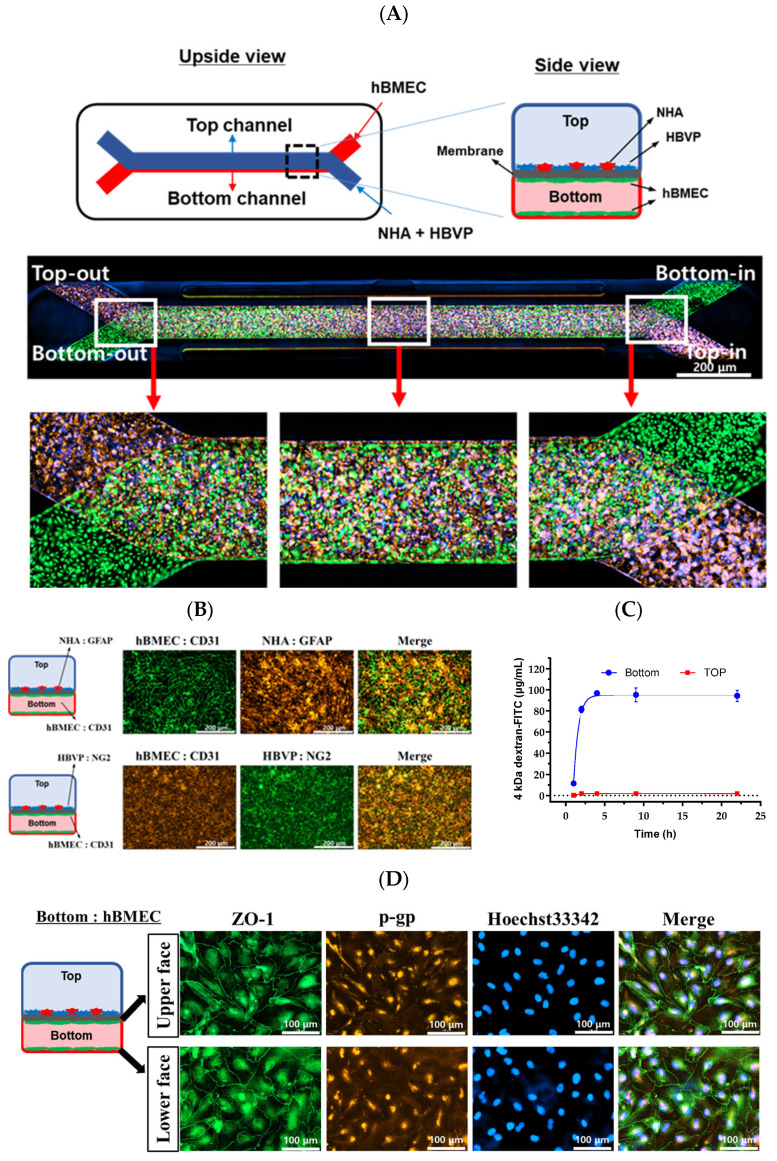
A two-channel microengineered chip had hBMECs (green) on all surfaces of the bottom channel, and NHAs (red) and HBVPs (blue) on the surface of the top channel (**A**). Each cell line was stained with specific markers. Endothelial cells (CD31), astrocytes (GFAP), and pericytes (NG2) (**B**). The bottom was perfused with 4 kDa dextran-FITC for 24 h (**C**). To confirm the formation of blood vessels by hBMEC, immunocytochemistry images were obtained of the junction protein ZO-1 (green) and transporter levels of expression of P-gp (red) in the bottom channel (**D**).

The design and construction of the BBB-on-a-chip were as reported previously [18,19]. The chip (Emulate, #OCK-12) was composed of two parallel microchannels (top; 1 × 1 mm, bottom; 1 × 0.2 mm) separated by a porous polydimethysiloxane (PDMS) membrane (diameter, 7 μm; spacing, 40 μm; thickness, 50 μm, resulting in 2% porosity over a surface area of 0.171 cm^2^ separating the two channels). The membrane was activated according to the manufacturer’s instructions and coated with the mixture of laminin (1 mg/mL), collagen IV (1 mg/mL), and fibronectin (1 mg/mL) in DPBS (Figure 1). Coated chips were incubated at 4 °C overnight, then at 37 °C for 1 h before seeding the cells. hBMEC cells (9 × 10^5^ cells/mL) were 20 µL seeded into the bottom channel of the chip. The chip was immediately inverted and incubated at 37 °C for 4 h. After 4 h, hBMECs were prepared as previously described and re-injected into the bottom channel to mimic the vascular structure, as shown in right panel of Figure 2A. HBVP and NHA were mixed at 0.04 × 10^5^ and 0.4 × 10^5^ cells/mL, respectively, and introduced 50 µL into the top channel (Figure 2A); the chip was in a static culture at 37 °C for 1 day and then connected to the PodTM Portable Module microfluidic pump at a flow bottom (30 μL/h)/Top (80 μL/h) for 1 day.

### 2.4. Confirmation of Cell Seeding

hBMECs were detached from the cell-culture plate and stained with CellTracker™ Green CMFDA dye (C2925, Invitrogen, Waltham, MA, USA). HBVPs and NHAs were detached from the cell-culture plate and stained with blue CMAC dye (C2110, Invitrogen) and orange CMTMR dye (C2927, Invitrogen), respectively. After 24 h, fluorescence images were obtained using the Lionheart FX Automated Microscope (BioTek Instruments, Winooski, VT, USA). These chips were used for the permeability test.

### 2.5. Immunofluorescence

Cells were fixed with 4% formaldehyde and blocked on the brain-on-a-chip in phosphate-buffered saline (PBS) containing 10% fetal bovine serum at 4 °C overnight. The primary antibodies were ZO-1, P-gp (1:200, 40–2200, MA5-13854, Thermo Fisher Scientific, Waltham, MA, USA), GFAP (1:200, 560298, BD pharmingen, NJ, USA), NG2 (1:200, ab129051, Abcam, Cambridge, UK), and CD31 (1:200, 303110, BioLegend, San Diego, CA, USA). Chips treated with corresponding Alexa Fluor-conjugated secondary antibodies (1:500, Abcam) were incubated in the dark for 2 h at room temperature. Chips were then washed in PBS. Cells were counterstained with the nuclear dye Hoechst 33342 and visualized using the Lionheart FX Automated Microscope (BioTek Instruments, Winooski, VT, USA).

### 2.6. Drug Treatment and Sampling

The permeability study was carried out 48 h after the seeding cells in the BBB-on-a-chip. Ten drugs (Table 1) were selected for BBB permeability analysis (CAF, CBZ, DES, LPM, CET, VIN, LP, NZD, DPZ, and SIM). For apical-basolateral (A to B) permeability analysis, the hBMEC media were diluted to the final concentration of 1 μM and treated in the bottom channel (apical, blood channel), and the media were collected at 1, 4, 9, and 24 h from the outlet reservoirs of the bottom and top (basolateral, brain channel), respectively (Figure 1). For basolateral-apical (B to A) permeability analysis, the NHA and HBVP (1:1) media were diluted to the final concentration of 1 μM and treated in the top channel, and the media were collected at 1, 4, 9, and 24 h from the outlet reservoirs of the bottom and top, respectively. We performed transport experiments for the basolateral-to-apical side and the apical-to-basolateral side.

### 2.7. LC-MS/MS Analysis

Fifteen microliters of blood-and-brain medium was mixed with 135 μL internal standard (IS) solution (5 ng/mL disopyramide in acetonitrile). The mixture was vortexed briefly and centrifuged at 15,000 rpm for 5 min at 4 °C. The supernatant (100 μL) was transferred to a sample vial for LC-MS/MS analysis using a liquid chromatograph (Agilent 1260) and 4000 Qtrap quadruple mass spectrometer (LC-MS/MS, AB Sciex, Foster City, CA, USA) analysis. Mobile phase A was 0.1% formic acid in water and mobile phase B was 0.1% formic acid in acetonitrile. Chromatographic separation was performed on a Luna C18 column (100 × 2 mm (i.d.) 3 μm, Phenomenex) with a Security Guard C18 guard column (4 mm × 20 mm (i.d.), Phenomenex) by gradient elution at a flow rate of 0.3 mL/min (Table 2). The mass spectrometer was operated in positive ion mode for all compounds, and the carryover was checked by the injection of a blank in between samples.

### 2.8. Data Analysis

Drug concentration at 0–1, 1–4, 4–9, and 9–24 h were expressed as the concentrations at 1, 4, 9, and 24 h as $C_{channel},\;C_{channel}$; the concentration of the top channel or bottom channel was calculated by adding the amount of drug measured in that interval to the amount of drug measured up to the previous time point, as seen in Equation (1).

(1) $$C_{t_{n},channel}=\frac{A_{t_{n-1,\;channel}}+C_{{(t}_{n}-t_{n-1}),channel}\cdot \left(t_{n}-t_{n-1}\right)\cdot channel\;flow\;rate}{t_{n}\cdot channel\;flow\;rate},$$

where $A_{channel}$ is the drug amount of the channel; $t_{n}$ is time point; $C_{{(t}_{n}-t_{n-1}),channel}$ is the concentration of drug pooled from $t_{n}$ to $t_{n-1}$; *channel flow rate*, fluid flow rate in channel;

The concentrations calculated after dosing the fluidic system were analyzed using Python (ver.3.9.16) to estimate the pharmacokinetic parameters of the drugs on the BBB-on-a-chip.

$C_{ss,\;channel}$, the concentration in the channel at the steady state, was estimated using a simple $E_{max}$ model Equation (2) with time (h) on the *x*-axis and concentration (ng/mL) on the *y*-axis.

(2) $$C_{channel}=\frac{C_{ss,\;channel}\;\cdot \;t}{{CT}_{50,\;channel}+t},$$

where ${CT}_{50,\;channel}$ is the time at a half steady state concentration of channel; *t*, time.

$K_{p}$, the partition coefficient, in the BBB-on-a-chip was calculated by dividing the calculated $C_{ss,\;brain}$ by the $C_{ss,\;blood}$ value of the drug passing through the bottom channel, as shown in Equation (3).

(3) Kp=Css, brainCss, blood

The apparent permeability ($P_{app}$) is calculated from the profile of the proposed simple $E_{max}$ model Equation (4). J represents the rate at which the amount of compound moves from the bottom channel to the top channel, which was calculated by determining the slope of the time-versus-amount curve at 0 h to the point at which a steady state (90% of $T_{ss}$) was reached [31]. $P_{app}$ can be calculated by dividing *J* by the surface area of the membrane (*SA*) separating the two channels and the initial concentration in the bottom channel.

(4) Papp=JMWSA·C0,blood ,J=dAbraindt,

where *MW* is molecular weight of each drug.

### 2.9. Statistical Analysis

Means were compared between and among groups using unpaired *t*-tests or one-way ANOVA followed by Tukey’s post hoc test. Data are expressed as means ± SD. A value of *p* < 0.05 is considered indicative of statistical significance.

## 3. Results

### 3.1. Schematic Diagram and Functional Validation of hBBB Models

For the microfluidic-based BBB test, we introduced hBMECs into the lower channel of Chip-S1, and astrocytes and pericytes into the upper channel under the same conditions as described in Section 2—“Materials and Methods”. To confirm the attachment of these cells within the chip, we observed fluorescence for each cell type. After 24 h of cell seeding, we confirmed that hBMEC (green) in the lower channel and astrocytes (red) and pericytes (blue) in the upper channel were uniformly attached (Figure 2A). To ensure the formation of tight junctions between the two channels in the BBB chip, we maintained a steady flow in each channel for an additional 24 h. Each cell line was stained with specific markers (Figure 2B). CD31 was specifically stained with hBMEC. The bottom channel’s hBMEC is co-stained with the top channel’s NHA. CD31 was clearly seen at the intercellular boundary of hBMEC. GFAP, specifically NHA, was shown in the top channel. In addition, the bottom channel’s hBMEC was co-stained with the top channel’s HBVP. CD31 was clearly seen at the intercellular boundary of hBMEC, and NG2 specifically staining HBVP was seen in the top channel. Subsequently, to verify the restricted non-selective movement of substances between the two channels, we treated the lower channel with 10 μg/mL dextran-FITC. We then collected medium from each channel’s outlet at different time points and measured the fluorescence (Figure 2C). The fluorescence in the lower channel treated with the substance was similar to the treated concentration, indicating that the medium containing dextran-FITC flowed out similarly after 1 h. In contrast, in the upper channel where dextran-FITC was not treated, the fluorescence of dextran-FITC was not observed for 22 h, confirming the well-formed tight junctions by the cultured hBMEC in the lower channel. Additionally, when performing immunostaining for the tight junction protein ZO-1, we confirmed that ZO-1 was well-arranged at the membrane of the cultured hBMEC in the lower channel (Figure 2D). These results confirm that the BBB chip used in this study is suitable for BBB permeability testing conditions and was subsequently used for BBB permeability experiments.

### 3.2. Assessment of hBBB Permeability Using Microfluidic Model

**Figure 3:**
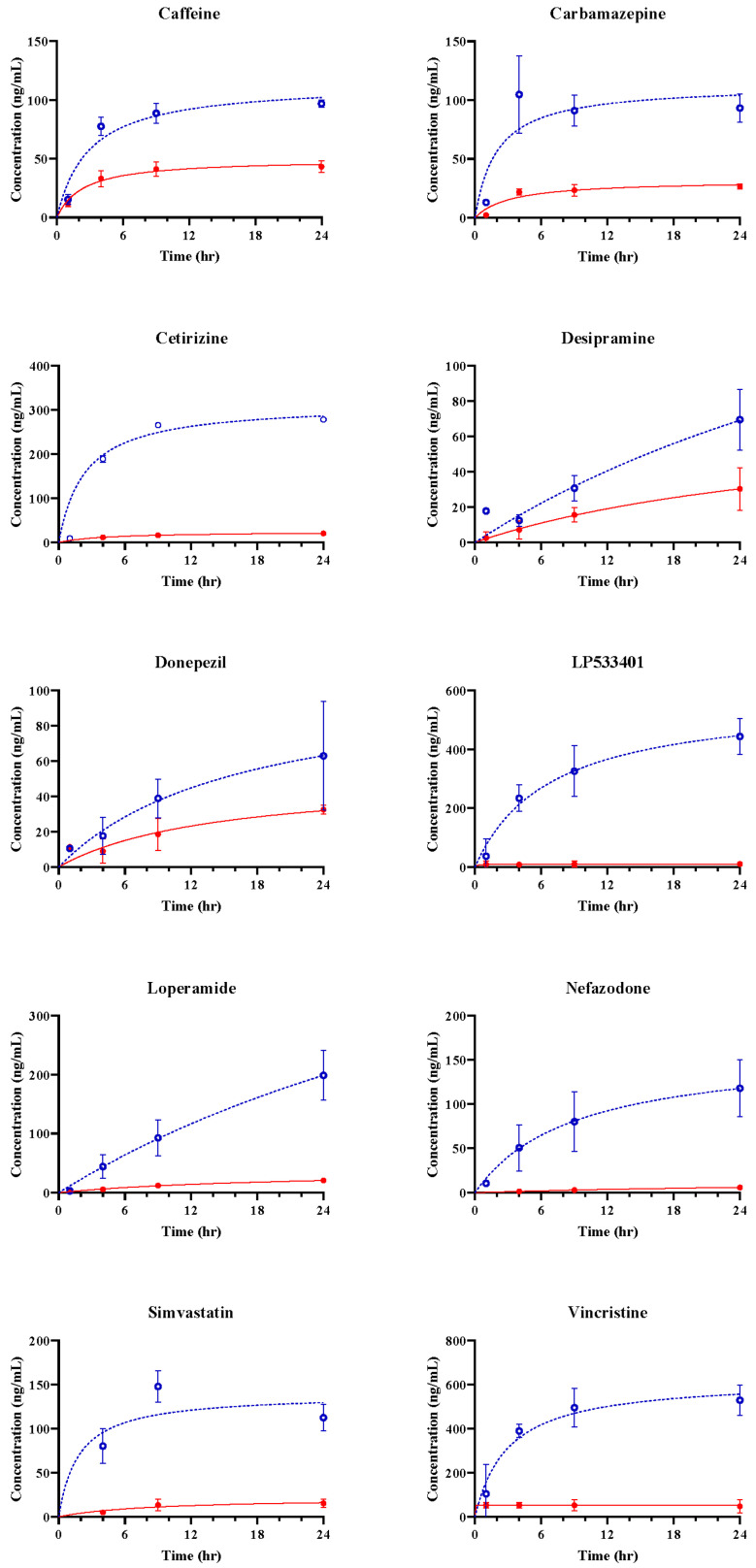
Observed and estimated drug concentration of the microfluidic system (apical to basolateral). Blue circles (ο) and red dots (●) represent the drug concentration in the blood channel and the brain channel, respectively. Each point is means ± SD (n = 3). Dashed and solid lines represent the expected concentrations in the blood and brain based on Equations (1) and (2). SD, standard deviation; n, the number of repetitions for completely separate individual experiments.

**Figure 4:**
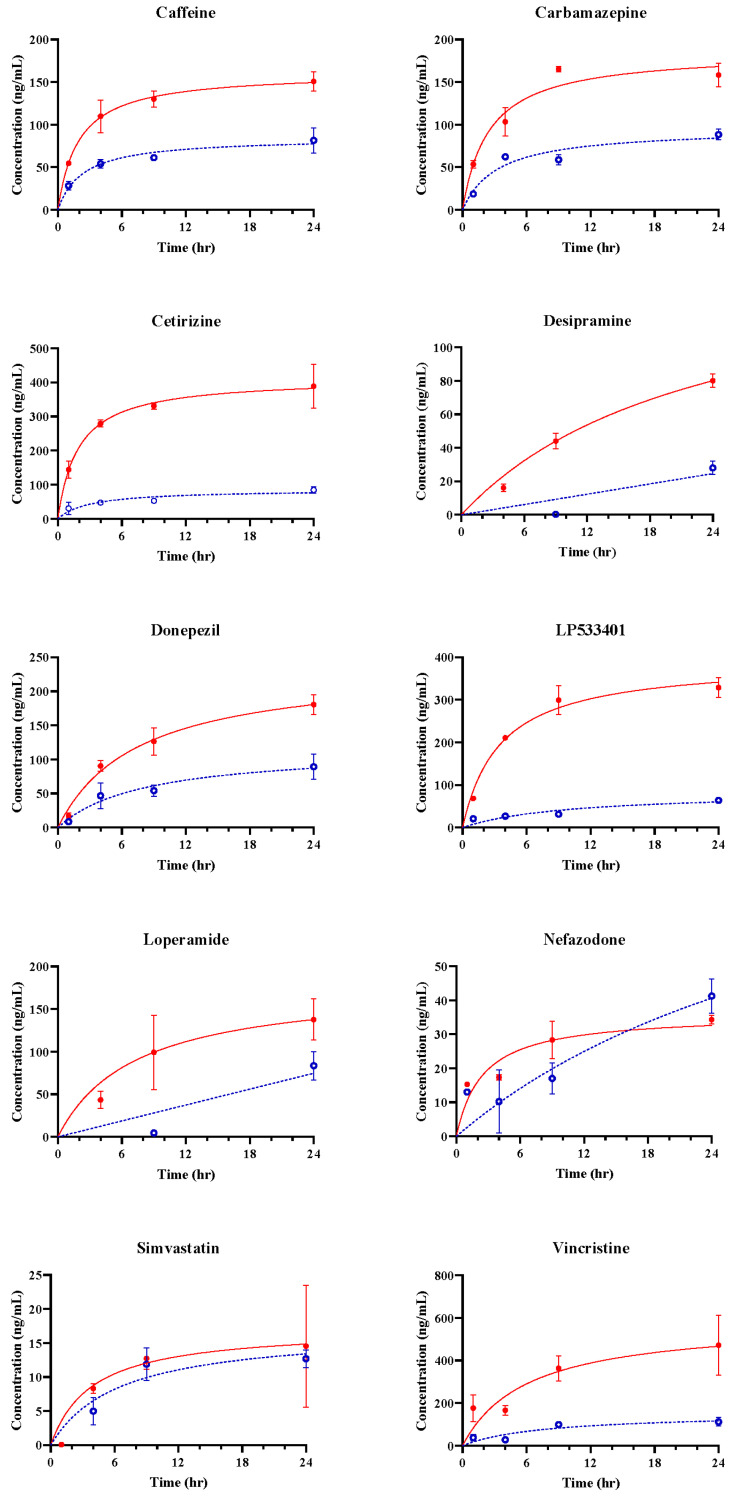
Observed and estimated drug concentration of the microfluidic system (basolateral to apical). Blue circles (ο) and red dots (●) represent the drug concentration in the blood channel and the brain channel, respectively. Each point is means ± SD (n = 3). Dashed and solid lines represent the expected concentrations in the blood and brain based on Equations (1) and (2). SD, standard deviation; n, the number of repetitions for completely separate individual experiments.

Passing through the vascular barrier is essential for drug transport. Therefore, we conducted permeability experiments on 10 different compounds with varying BBB permeabilities using a BBB-mimicking microfluidic chip. To analyze concentration changes, we performed LC-MS/MS. Samples were collected from the blood and brain channels during 0–1, 1–4, 4–9, and 9–24 h, and concentrations at 1, 4, 9, and 24 h were calculated using Equation (1). The concentrations at each channel, based on the time profiles, are depicted as blue circles (ο) and red dots (●). The predicted concentrations for each channel, calculated using Equation (2), are represented by blue dashed and red solid lines (Figure 3 and Figure 4). CAF, used as a positive control for simple diffusion across the BBB, demonstrated high permeability in the apical to basolateral drug permeability test (Figure 3). Additionally, LPM, known as a substrate of P-glycoprotein (P-gp), exhibited high permeability in the basolateral to apical drug permeability test (Figure 4).

### 3.3. In Vitro BBB Models for Drug Transport Screening

The concentrations of 10 model drugs were calculated at 1, 4, 9, and 24 h after dosing in the fluidic BBB-on-a-chip system (Figure 3 and Figure 4). The $t_{plateau,\;channel}$, plateau time for each channel, was calculated as the time to reach the predicted 90% Css (Table 3 and Table 4).

Most drugs did not reach plateau during the time observed in the experiments, but several drugs (e.g., caffeine, Lp533401, and vincristine) reached plateau in the brain channel (Table 4).

$K_{p}$ was calculated using the ratio of Css values in the two channels. Differences in $K_{p}$ can differ between the BBB-on-a-chip and that in vivo [32].

The $P_{app}$ values were calculated using the slope starting from at 0 h to the calculated brain plateau time (Table 4).

**Figure 5:**
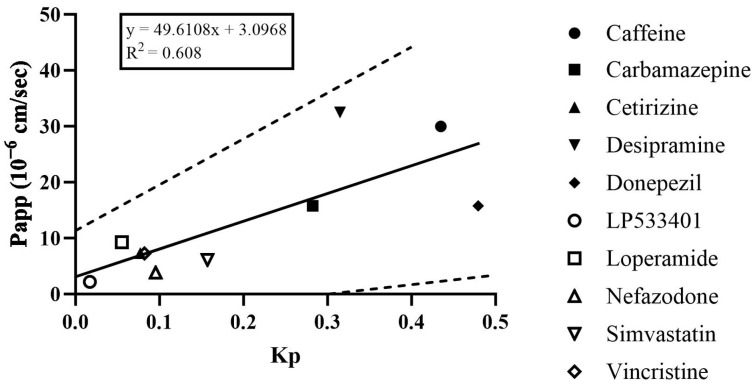
Linear regression of the relationship between parameters of the microfluidic system. Solid line and dashed lines represent the regression line and the 95% confidence interval.

The $K_{p}$ values were 0.0170 and 0.4794, and $P_{app}$ values were 2.1693 to 32.4598. Regardless of the level of BBB permeability, linear regression of the $P_{app}$ and $K_{p}\;$ values showed a positive coefficient of determination of 0.608, and the values for all the compounds were within 95% confidence intervals (Figure 5). This indicates a proportional correlation between the $K_{p}$ and $P_{app}$ values. Therefore, obtaining $P_{app}$ and $K_{p}$ using concentrations calculated by extending the data obtained at discrete times to cumulative data in an organ chip where the material continuously flows with the medium in different channels is an effective way to evaluate the permeation of the organ chip.

## 4. Discussion and Conclusions

The BBB is a critical consideration in the development of drugs for neurological diseases. Recent advancements in microfluidic technology have greatly improved BBB-on-a-chip models, enabling them to recreate the brain microenvironment and physiological responses. Compared to 2D Transwell technology, microfluidic BBB-on-a-chip models can simulate the in vivo environment, enabling prediction of the pharmacokinetics in the BBB [33,34]. In contrast to the traditional 2D Transwell approach, microfluidic systems allow perfused culture of brain endothelial cells, which is essential for proper lumen formation and expression and localization of junction proteins (Figure 2D). Microfluidic flow is generated by the Zoe-CM1 culture module, allowing medium flow from inlet to outlet. The barrier function of brain endothelial cells is reportedly enhanced by high shear stress due to upregulation of junction proteins [35,36].

We developed a microfluidic chip-based human-on-a-chip model for estimating the BBB permeability of drug candidates. We used human-derived cells to mimic the structure and function of the human BBB, and measured the BBB permeability of caffeine (CAF), carbamazepine (CBZ), desipramine (DES), loperamide (LPM), cetirizine (CET), vincristine (VIN), nefazodone (NZD), donepezil (DPZ), simvastatin (SIM), and LP533401 (LP). Some chemicals used in this study were tested in the previous BBB permeability study using rodents and zebrafish. This study did not show the same absolute value of $K_{p}$ due to differences in the experimental systems [32]. It is necessary that an experiment using BBB-on-a-chip models built based on various chemicals as well as a thorough search of the literature are needed.

Our model showed that CAF and CBZ have high permeabilities, and LP and CET showed low permeability. These results are consistent with previous studies using animal or cell-culture models [25,27,37,38]. CAF is a well-known positive control for simple diffusion across the BBB, and it showed high permeability in both blood and brain channels in our model. CBZ is an antidepressant that can cross the BBB by passive diffusion or active transport. LP is a TPH1 inhibitor and CET is a second-generation histamine-H(1)-receptor antagonist with poor BBB penetration. SIM is an antihyperlipidemic drug with low molecular weight but poor BBB penetration due to efflux by P-gp [39]. Further research will be necessary in the future, but if various membrane proteins related to drug transport are well-expressed in the BBB chip, we consider the microfluidic BBB-on-a-chip model as a testing method that could potentially replace traditional cellular or animal models.

Our model has several advantages over current methods for predicting BBB permeability. First, our model uses human-derived cells to mimic the human BBB, thereby overcoming any species differences. Second, it incorporates three cell types in a dynamic and tunable microenvironment, thus reproducing the complexity and diversity of the human BBB better than cell-cultures. Third, it enables quantitative measurement of BBB permeability by LC-MS/MS, which provides more reliable data than qualitative methods such as immunofluorescence staining. Fourth, it is more cost-effective and ethical than animal models, because it requires less time, space, and resources.

Tissue chips with microfluidic systems are unable to explain the distribution of drugs in the body, including the systemic circulation. However, in this study, we addressed this limitation by representing discrete data as accumulated data and calculating concentration values over an extended period. This allowed us to evaluate drug permeability in the tissue chip and derive permeability trends similar to previous animal and cell-based experiments. We also confirmed a general correlation between $P_{app}$ and $K_{p}$. The proposed method for evaluating drug permeability in tissue chips, used in this study, involved conducting permeability assessments at multiple time points, similar to in vitro permeability experiments (e.g., PAMPA, Caco-2), which have been commonly used to assess the permeability of compounds [40,41,42]. As a result, this research in tissue chips, which had been relatively less explored compared to toxicity and pharmacological studies, could lead to more effective drug evaluations in the field of pharmacokinetics.

This study had several limitations that need to be addressed in future work. First, our model does not include other factors that may affect BBB permeability, such as inflammation, oxidative stress, and neurodegeneration. These factors may alter the expression or function of BBB components, such as tight junctions or transporters, and influence drug delivery to the brain [16]. Second, the model does not account for the regional heterogeneity of the human BBB, which can also be altered under pathology. For example, some brain regions have higher or lower expression of P-gp or other efflux transporters, which can affect the drug sensitivity. Third, it does not consider the interactions between drugs and components of the blood or brain, such as plasma proteins and neurotransmitters. Such interactions may affect the free fraction or distribution of drugs in the blood or brain, and modulate their pharmacological effects. Fourth, it does not reflect the pharmacokinetics or pharmacodynamics of drugs in vivo, which may depend on factors such as metabolism or clearance. These factors can affect the bioavailability or efficacy of drugs in the systemic circulation and target tissues.

In conclusion, we developed a novel microfluidic chip-based human-on-a-chip model for evaluation of the BBB permeability of drugs. We used human-derived cells to mimic the human BBB and measured the BBB permeability of 10 compounds. We compared of our results with those of animal and cell-culture models, and demonstrated the advantages and validity of our model. Our model represents a new paradigm for BBB permeability prediction and could improve the efficiency and safety of drug development.

## Figures and Tables

**Table 1:** Information for 10 compounds used to study permeability of the BBB-on-a-chip.

Chemicals	Target	Reference
Caffeine (CAF)	CNS stimulant	[20]
Carbamazepine (CBZ)	Anticonvulsant drug	[21,22]
Desipramine (DES)	Anti-depressant	[23]
Loperamide (LPM)	P-gp substrate	[24]
Cetirizine (CET)	Antihistamine drug	[25]
Vincristine (VIN)	Anticancer drug	[26]
LP533401 (LP)	Tryptophan hydroxylase 1 inhibitor	[27]
Nefazodone (NZD)	Serotonin antagonist	[28]
Donepezil (DPZ)	Cholinesterase inhibitor	[29]
Simvastatin (SIM)	Antihyperlipidemic drug	[30]

**Table 2:** LC-MS/MS parameter of test chemicals.

Chemicals	MRM Transition		Collision Energy
	Precursor Ion [M + H]^+^	Product Ion	
Caffeine (CAF)	195.1	138.0	18.0
Carbamazepine (CBZ)	237.1	194.1	14.0
Desipramine (DES)	266.8	72.2	31.0
Loperamide (LPM)	477.1	266.1	25.0
Cetirizine (CET)	389.2	201	25.0
Vincristine (VIN)	825.4	807.4	539
LP533401 (LP)	527.1	253.0	30.0
Nefazodone (NZD)	281.3	86	14.0
Donepezil (DPZ)	311	143	10.0
Simvastatin (SIM)	345.2	143	18.0

**Table 3:** Summary of kinetic parameters of the microfluidic system.

Chemicals	${CT}_{50,blood}$ (h)	$C_{ss,blood}$ (ng/mL)	${CT}_{50,brain}$ (h)	$C_{ss,brain}$ (ng/mL)	$K_{p}$ (Brain/Blood)
Caffeine	2.91	114.47	2.39	49.79	0.4349
Carbamazepine	1.98	112.88	3.27	31.86	0.2823
Cetirizine	2.37	314.91	4.46	24.27	0.0771
Desipramine	57.44	234.83	34.47	73.93	0.3148
Donepezil	16.55	106.64	14.43	51.12	0.4794
LP533401	6.80	573.66	0.16	9.77	0.0170
Loperamide	58.46	684.09	20.04	37.75	0.0552
Nefazodone	9.01	161.89	37.79	15.43	0.0953
Simvastatin	1.83	139.52	8.72	21.91	0.1570
Vincristine	2.99	626.20	0.00	51.41	0.0821

${CT}_{50,channel}$, time at half steady state concentration of channel; $C_{ss,channel}$ the concentration of the channel; ($K_{p})$, the partition coefficient.

**Table 4:** Linear regression analysis results.

Chemicals	$C_{plateau,brain}$ (ng/mL)	$t_{plateau,brain}$ (h)	$P_{app}$ $\left(10^{-6}cm/s\right)$
Caffeine	44.8098	21.52	29.9873
Carbamazepine	28.6749	29.41	15.7719
Cetirizine	21.8407	40.16	7.2984
Desipramine	66.5383	310.22	32.4598
Donepezil	46.0063	129.86	15.7542
LP533401	8.7886	1.42	2.1693
Loperamide	33.9736	180.32	9.2550
Nefazodone	13.8908	340.10	3.8408
Simvastatin	19.7183	78.50	6.1221

$C_{plateau,brain}$, concentration at plateau time of brain channel; $t_{plateau,\;brain}$, plateau time for brain channel; $P_{app}$, the apparent permeability.

## Data Availability

The data presented in this study are available in this article.
